# Bacterial–Fungal Co-Occurrence in the Porcine Gut Microbiome Is Associated with Distinctive Meat Flavor Profiles in Indigenous Congjiang Xiang Pigs

**DOI:** 10.3390/vetsci13070721

**Published:** 2026-07-22

**Authors:** Kang Yang, Li Lin, Chunying Sun, Guoxi Sun, Qiuyue Li, Xiaoyu Li, Chuntao Long, Qiaowen Tang, Xianrong Shi, Jiapei Wang, Hailiang Xin, Baichuan Deng, Jiada Yang

**Affiliations:** 1 School of Life and Health Science, Kaili University, Kaili 556011, China; yangkang@kluniv.edu.cn (K.Y.); 15085286854@163.com (L.L.); 13595520703@163.com (C.S.); 17628312939@163.com (G.S.); 18185585150@163.com (Q.L.); 19315423801@163.com (X.L.); 18308651706@163.com (C.L.); 19885073498@163.com (Q.T.); m18785541244@163.com (X.S.); 2 Department of Biological and Environmental Engineering, Qian Dongnan Nationalities Polytechnic, Kaili 556011, China; wjp010101@163.com (J.W.); hailianghebau@163.com (H.X.); 3 Guangdong Provincial Key Laboratory of Animal Nutrition Control, College of Animal Science, South China Agricultural University, Guangzhou 510642, China

**Keywords:** Congjiang Xiang pig, bacterial–fungal co-occurrence, meat flavor, gut microbiota, multi-omics

## Abstract

Meat flavor significantly influences consumer preference and market value, particularly for indigenous pig breeds renowned for distinctive sensory characteristics. While traditional research has focused on genetic factors and feeding regimens, emerging evidence suggests that gut microbiota plays a crucial role in meat quality attributes. However, the specific contribution of bacterial–fungal co-occurrence to meat flavor formation remains largely unexplored. This study aimed to characterize the associations between intestinal bacterial–fungal co-occurrence networks and the muscle flavor-related metabolite profiles of CX pigs, using an integrated multi-omics approach. Twenty male pigs (10 CX and 10 LAN, 12 months old) were subjected to comprehensive analyses, including meat quality evaluation, electronic nose analysis, 16S and 18S rRNA sequencing, and untargeted metabolomics. CX pigs exhibited significantly superior meat quality characteristics, including higher moisture content (*p* < 0.001), fat content (*p* = 0.008), and meat color scores (*p* < 0.001). Electronic nose analysis revealed significantly higher response values across all ten aroma sensors in CX pigs (*p* < 0.001), with the most pronounced differences observed in sensors detecting sulfur compounds and organic compounds. Untargeted metabolomics identified 40 differential metabolites, with 27 up-regulated in CX pigs, including key flavor compounds such as glycocholic acid, isorhamnetin, and pantothenic acid. Microbiome analysis demonstrated significantly higher bacterial alpha diversity in CX pigs (*p* < 0.05), with enrichment of beneficial bacteria, including *Rikenellaceae_RC9_gut_group*, *Prevotellaceae_UCG_003*, and *Phascolarctobacterium*, while fungal communities showed enrichment of *Candida_Lodderomyces_clade*. Correlation network analysis revealed that *Rikenellaceae_RC9_gut_group* demonstrated strong positive correlations with flavor compounds (r = 0.575 for isorhamnetin, r = 0.535 for pantothenic acid, *p* < 0.001) and all electronic nose responses (r = 0.434–0.691, *p* < 0.001). Bacterial–fungal co-occurrence networks showed synergistic relationships, with *Rikenellaceae_RC9_gut_group* positively correlated with *Candida_Lodderomyces_clade* (r = 0.711, *p* < 0.001) while exhibiting antagonistic relationships with *Piromyces* (r = −0.714, *p* < 0.001). These findings offer novel insights for developing microbiome-targeted strategies to enhance meat quality in pig production systems.

## Simple Summary

Local pig breeds are prized for the rich, distinctive taste of their meat, but scientists still do not fully understand why their meat tastes so good. One possible reason is the vast community of tiny living organisms, such as bacteria and fungi, that live inside the pig’s gut. These microbes help digest food and produce substances that may travel to the muscle and shape its flavor. In this study, we compared a traditional Chinese village pig, the Congjiang Xiang pig, with a common commercial breed. We measured the taste and smell of the cooked meat, the chemical compounds inside the muscle, and the mix of bacteria and fungi living in the gut. We found that the village pigs had meat with more intense aroma and more flavor-related compounds, and their guts contained certain helpful bacteria and fungi that appeared to work together and were closely linked to these desirable flavor substances. These results suggest that the gut community may help create tasty meat. In the future, farmers might be able to encourage these beneficial gut organisms through feeding or breeding, helping ordinary farmed pigs produce meat that is just as flavorful as that of treasured local breeds.

## 1. Introduction

Meat flavor is a critical quality attribute that significantly influences consumer preference and market value, particularly for indigenous pig breeds renowned for their distinctive sensory characteristics [1]. The Congjiang Xiang (CX) pig, an indigenous breed from southwest China, is highly valued for its exceptional meat quality and unique flavor profile that distinguishes it from commercial breeds [2,3]. Traditionally, research on meat flavor formation has concentrated on genetic factors, feeding regimens, and intramuscular fat content as primary determinants of flavor development [4]. However, emerging evidence suggests that the gut microbiota plays a crucial role in shaping host metabolism and potentially influences meat quality attributes, including flavor [5].

The intestinal microbiota represents a complex ecosystem comprising bacteria, fungi, viruses, and archaea that collectively impact host physiology through multiple pathways [6]. Recent investigations have demonstrated that intestinal bacteria can modulate lipid metabolism and contribute to the formation of flavor-associated compounds through the production of metabolites, which serve as precursors for volatile flavor compounds [7]. While bacterial communities have been the focus of most gut microbiome studies, fungi constitute an important yet often overlooked component of the intestinal ecosystem. Fungi possess distinct metabolic capabilities and produce unique secondary metabolites that may influence host metabolism and potentially contribute to flavor development in meat-producing animals [8]. Despite growing interest in microbiome–host interactions, the specific contribution of bacterial–fungal co-occurrence to meat flavor formation remains largely unexplored. Most studies have examined bacterial and fungal communities in isolation, neglecting their potential synergistic or antagonistic interactions [9,10]. These microbial interactions may create unique metabolic networks that influence the generation of flavor precursors and volatile compounds in muscle tissue. In indigenous breeds like the CX pig, which have co-evolved with distinct microbial communities under traditional rearing conditions, these bacterial–fungal interactions may be particularly relevant to the development of their characteristic flavor profiles [11].

Current research on meat flavor has primarily employed targeted approaches focusing on specific metabolic pathways or compound classes. Integration of multi-omics technologies, including metagenomics, metabolomics, and flavoromics, offers a more comprehensive understanding of the complex relationship between gut microbiota and flavor formation [12,13]. Furthermore, there is limited information regarding the mechanistic pathways through which gut microbial communities regulate the generation of volatile flavor compounds in indigenous pig breeds such as the CX pig [14,15]. To address these knowledge gaps, the present study aims to characterize the associations between intestinal bacterial–fungal co-occurrence networks and the muscle flavor-related metabolite profiles of CX pigs, using an integrated multi-omics approach. Rather than establishing causality or mechanism, we systematically describe microbiome composition, muscle metabolites, and volatile/odor profiles and identify correlation structures that may generate hypotheses for future functional testing.

## 2. Materials and Methods

### 2.1. Animal Ethics Statement

All experimental procedures were approved by the Academic Committee of Kaili University (Approval No: 202402).

### 2.2. Animals and Sample Collection

Ten Congjiang Xiang pigs (male, 12 months old) and 10 Landrace (male, 12 months old) were used in this study. All male pigs were castrated and raised at similar altitudes and shared comparable natural climatic conditions in Congjiang County, Qiandongnan Miao and Dong Autonomous Prefecture, Guizhou Province. Both groups of pigs were fed the same basal diet, formulated from 65% corn, 25% tofu residue (okara), and 10% rice bran, together with vegetables. All ingredients were cooked together before feeding, and the diet was supplemented with sodium chloride (NaCl) and dibasic calcium phosphate (CaHPO_4_). No antibiotics or antifungal agents were included in the diet. Based on tabulated ingredient values, the basal diet provided approximately 13% crude protein and a digestible energy level of approximately 3.3 Mcal/kg (13.8 MJ/kg). All pigs were housed in large group pens, given ad libitum access to feed and water, and raised over a three-month feeding period.

At the age of 12 months, pigs were weighted with an average body weight of 74.95 ± 4.68 kg (CX) and 189.95 ± 12.95 kg (LAN). Before slaughter, pigs were fasted for 12 h with free access to water, and jugular blood (10 mL) was collected and centrifuged (3000× *g*, 10 min, 4 °C) for serum. Pigs were euthanized, and within 30 min postmortem, *Longissimus dorsi* (LD) samples were collected between the 10^th^ and 12th ribs and snap-frozen; colonic contents (5–10 g) were then collected aseptically. All samples were stored at −80 °C until analysis.

### 2.3. Meat Quality Evaluations

The pH values of LD muscle were measured at 45 min postmortem (pH_45min_) using a portable pH meter (Testo 205, Testo AG, Titisee-Neustadt, Germany). Meat color and marbling scores were evaluated by three trained panelists according to the Technical Code of Practice for Pork Quality Assessment of the People’s Republic of China (NY/T 821-2019) [16]. Meat color was scored on a 1–6-point scale and marbling on a 1–6-point scale using the standard reference cards specified in NY/T 821-2019, and the mean score of the three panelists was used for statistical analysis.

The volatile flavor compounds of cooked LD muscle were analyzed using an electronic nose (PEN3.5, AIRSENSE, Schwerin, Germany), which contains 10 metal-oxide semiconductor sensor arrays; the name and performance description of each sensor are shown in Table 1. For sample pretreatment, 3.0 g of cooked LD muscle was minced and accurately weighed into a 20 mL airtight headspace vial, which was then sealed and equilibrated at 25 °C for 30 min to generate headspace volatiles. The measurement parameters were as follows: sample injection (air) flow rate of 300 mL/min, carrier (zero) gas flow rate of 300 mL/min, measurement/detection time of 120 s, sensor cleaning (flushing) time of 100 s, and auto-zeroing time of 5 s. The stable sensor response values recorded between 118 and 120 s were extracted for subsequent data analysis. Each sample was measured in triplicate.

### 2.4. Nutritional Value

Muscle samples were determined for dry matter according to AOAC methods. Crude protein and acid-hydrolyzed fat were determined using AOAC with the help of a Kjeldahl method with a semi-automatic Kjeldahl apparatus (VAPODEST 200, C. Gerhardt GmbH & Co. KG, Königswinter, Germany) and a fatty analyzer (FT640, Grand Analytical Instrument Co., Ltd., Guangzhou, China).

### 2.5. Determination of Biochemical Indices 

Serum and LD muscle concentrations of total cholesterol (TC), triglycerides (TG), high-density lipoprotein cholesterol (HDL-C), and low-density lipoprotein cholesterol (LDL-C) were determined using commercial kits (Nanjing Jiancheng Bioengineering Institute, Nanjing, China).

### 2.6. Untargeted Metabolomics Analysis

Untargeted metabolomics analysis of LD muscle was processed as described previously with minor modifications [17]. The UPLC-Orbitrap-MS/MS system from Thermo Fisher Scientific (Q-Exactive Focus, Thermo Fisher Scientific, Waltham, MA, USA) served as an untargeted metabolomic approach and was used to detect the LD muscle metabolic profiles. The PCA and orthogonal partial least squares discriminant analysis (OPLS-DA) were done with the SIMCA-P 14.1 software (Umetrics, Umea, Sweden). Response permutation testing (RPT) was performed to test the accuracy of OPLS-DA models. In addition, variable importance in the projection (VIP) was computed in the OPLS-DA model, and *p*-value was computed using an unpaired Student’s *t*-test. The metabolites with |log2FC| > 1, VIP > 1, and *p* < 0.05 were deemed as the differential metabolites. The KEGG database was applied to functionally annotate these differential metabolites, which were further mapped to the KEGG pathway database using the MetaboAnalyst 5.0 (https://www.metaboanalyst.ca).

### 2.7. 16S rRNA Gene Sequencing

Microbial DNA was extracted from colonic contents using the QIAamp Fast DNA Stool Mini Kit (Qiagen, Hilden, Germany). The V3-V4 hypervariable region of the bacterial 16S rRNA gene was amplified with the primers 338F/806R (CCTAYGGGRBGCASCAG, GGACTACNNGGGTATCTAAT), and then sequenced on an Illumina NovaSeq PE250 platform (Illumina, San Diego, CA, USA) with 250 bp paired-end reads. Raw reads were demultiplexed, quality-filtered, and denoised using the DADA2 pipeline in QIIME2 to obtain amplicon sequence variants (ASVs). Taxonomic assignment of ASVs was performed using a naive Bayes classifier trained on the SILVA database (release 138). Alpha diversity indices (Observed_ASV, Ace, Chao, Shannon, Simpson, and Goods_coverage) and beta diversity (Principal Coordinate Analysis, PCoA; Unweighted Pair-group Method with Arithmetic Mean, UPGMA) were calculated using the diversity plugin in QIIME2. Linear discriminant analysis effect size (LEfSe) was used to identify differentially abundant bacterial taxa between breeds, with an LDA score cutoff of 4 and *p*-value cutoff of 0.05.

### 2.8. 18S rRNA Gene Sequencing

The eukaryotic microbial community in colonic contents was analyzed by 18S rRNA gene sequencing. The 18S rRNA gene was amplified using the primers FR1/FF390 (GCGGTAATTCCAGCTCCAA, AATCCRAGAATTTCACCTCT), which target the V4 hypervariable regions. PCR products were purified, quantified, and sequenced on the Illumina NovaSeq PE250 platform with 250 bp paired-end reads. Raw reads were quality-filtered using QIIME2 and assembled into ASVs using the DADA2 plugin. Taxonomic assignment of ASVs was performed using the SILVA database (release 138). Alpha diversity indices (Observed_ASV, Ace, Chao, Shannon, Simpson, and Goods_coverage) and beta diversity (PCoA, UPGMA) of the eukaryotic microbial community were calculated. Differential abundance analysis between breeds was conducted using LEfSe with an LDA score cutoff of 4 and *p*-value cutoff of 0.05.

### 2.9. Statistical Analysis

Data were presented as mean ± standard deviation (SD). Comparisons between groups were performed by Student’s *t*-test or Mann–Whitney U test depending on data normality. Spearman’s correlation analysis was used to assess the relationships between microbial taxa, metabolites, and flavor compounds. Correlation networks were visualized using the OmicStudio tools at https://www.omicstudio.cn/tool (accessed on 10 December 2025). Significance was defined as *p* < 0.05.

## 3. Results

### 3.1. Meat Quality Parameters

Table 2 presents meat quality parameters of Congjiang Xiang (CX) and Landrace (LAN) pigs. CX pigs had significantly higher moisture and lower dry matter content compared to LAN pigs (*p* < 0.001). Fat content was also significantly higher in CX pigs than in LAN pigs (*p* = 0.008). In contrast, protein content was significantly lower in CX pigs compared to LAN pigs (*p* = 0.042). No significant difference was observed in pH_45min_ values between CX and LAN pigs. Meat color score, an important indicator of visual appeal, was significantly higher in CX pigs than in LAN pigs (*p* < 0.001).

### 3.2. Flavor Characteristics

To evaluate the flavor profiles of meat from CX and LAN pigs, electronic nose analysis was conducted, and the results are presented in Table 3. All ten aroma sensors (W1C/R(1) through W3S/R(10)) showed significantly higher relative response values in CX pigs compared to LAN pigs (*p* < 0.001). The most pronounced differences were observed in W1S/R(6) (5.033/2.270 = 2.217), W2W/R(9) (4.804/1.604 = 2.995), and W1W/R(7) (4.382/1.338 = 3.275).

**Figure 1:**
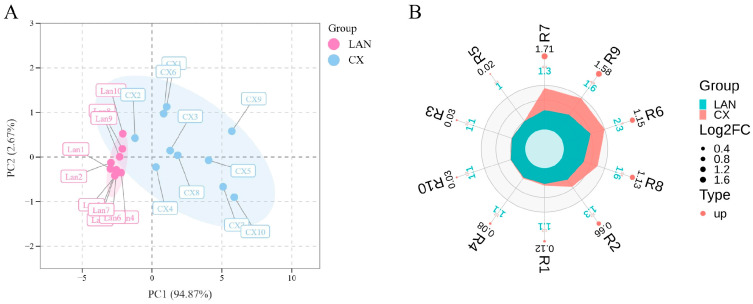
Flavor characteristics in CX and LAN pigs. (**A**) Principal component analysis (PCA) of electronic nose data between CX and LAN pigs. Blue dots represent CX pig samples, pink dots represent LAN pig samples. (**B**) Radar plot comparing flavor profiles across all ten aroma sensors (R1–R10) between CX and LAN pigs. The red area represents CX pigs, the teal area represents LAN pigs. The larger radar plot area demonstrates the more intense and complex flavor profile. LAN, Landrace; CX, Congjiang Xiang.

The principal component analysis (PCA) of the electronic nose data is presented in Figure 1. The PCA plot clearly demonstrates distinct clustering of CX and LAN samples (Figure 1A), indicating fundamentally different flavor profiles between the two breeds. The first two principal components (97.54%) explained a large portion of the total variance, with samples from each breed forming tight, non-overlapping clusters. This pattern suggests that the flavor characteristics of meat from CX pigs are consistently and distinctively different from those of LAN pigs. The radar plot (Figure 1B) further illustrates the comprehensive flavor profile differences between the two breeds. CX pigs exhibited markedly higher values across all measured aroma parameters compared to LAN pigs. The larger area covered by the CX pig profile in the radar plot visually demonstrates the more intense and complex flavor profile of meat from this indigenous breed.

### 3.3. Biochemical Parameters 

Biochemical parameters in serum and LD muscle were analyzed to evaluate the differences in lipid metabolism between CX and LAN pigs (Table 4). The results revealed significant differences in serum lipid profiles between the two breeds. CX pigs exhibited significantly higher serum total cholesterol (TC) levels compared to LAN pigs (*p* = 0.002). Similarly, serum high-density lipoprotein cholesterol (HDL-C) and low-density lipoprotein cholesterol (LDL-C) concentrations were also significantly elevated in CX pigs (*p* = 0.024 and *p* = 0.002, respectively). In contrast, serum triglyceride (TG) levels were significantly lower in CX pigs than in LAN pigs (*p* = 0.004). When examining the lipid profiles of the LD muscle, no significant differences were observed between breeds for TG and HDL-C concentrations. However, TC content tended to be lower in CX pigs compared to LAN pigs (*p* = 0.089). Additionally, LDL-C content in LD muscle was significantly lower in CX pigs compared to LAN pigs (*p* = 0.002).

### 3.4. Untargeted Metabolomics Profiling

**Figure 2:**
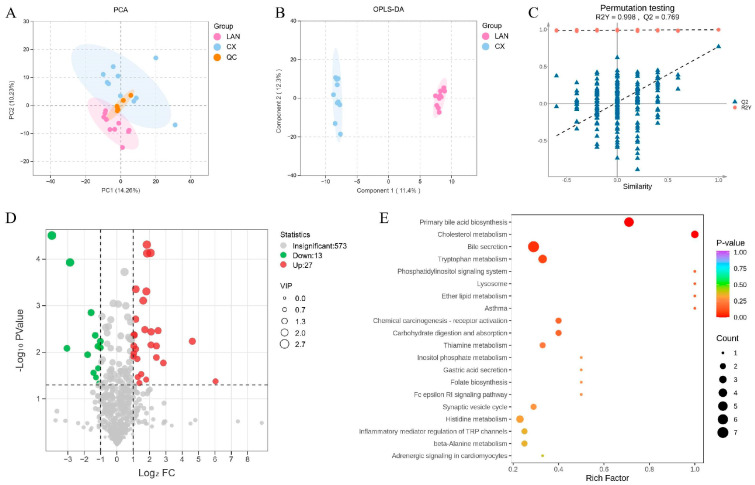
Untargeted metabolomics of LD muscle in CX and LAN pigs. (**A**) Principal component analysis (PCA). (**B**) Orthogonal partial least squares discriminant analysis (OPLS-DA) plot and (**C**) response permutation testing. (**D**) Volcano plot. (**E**) KEGG metabolic pathways enrichment analysis. LAN, Landrace; CX, Congjiang Xiang; QC, quality control sample.

To comprehensively investigate the metabolomic differences in meat between CX and LAN pigs, untargeted metabolomics analysis was performed on LD muscle samples, with results presented in Figure 2. The PCA of the untargeted metabolomics data (Figure 2A) revealed distinct separation between CX and LAN pig samples, with an additional quality control (QC) sample group clustered closely together, indicating good analytical stability. The first two principal components explained 24.49% of the total variance (PC1: 14.26%, PC2: 10.23%), with the CX and LAN samples forming largely non-overlapping clusters.

To further discriminate between the metabolite profiles, orthogonal partial least squares discriminant analysis (OPLS-DA) was performed (Figure 2B). The OPLS-DA model demonstrated excellent separation between CX and LAN groups, with the first two components explaining 11.4% and 12.3% of the variance, respectively. The model showed good predictive power with *R*^2^*Y* = 0.998 and *Q*^2^ = 0.769, confirming significant differences in muscle metabolite composition between the two breeds. The model validation through response permutation testing (Figure 2C) showed that the original model was valid and robust, with significantly higher *R*^2^ and *Q*^2^ values than those of the permuted models, indicating that the observed separation was not due to overfitting but reflected genuine biological differences.

The volcano plot analysis (Figure 2D) identified a total of 40 significantly differential metabolites between CX and LAN pigs, with 27 up-regulated (shown in red) and 13 down-regulated (shown in green) in CX pigs compared to LAN pigs (|log2FC| > 1, VIP > 1, and *p* < 0.05). Among the up-regulated metabolites in CX pigs were several flavor-related compounds, including glycocholic acid, cholic acid, 13-l-hydroperoxylinoleic acid, isorhamnetin, diosmin, poncirin, 5-(2-hydroxyethyl)-4-methylthiazole, sucrose, ribose 1-phosphate, pantothenic acid, hippuric acid, and genipin, which contribute to meat taste and aroma, and the down-regulated metabolite was malondialdehyde in CX pigs compared to LAN pigs (Table 5).

KEGG pathway enrichment analysis (Figure 2E) revealed that the differential metabolites were significantly enriched in multiple metabolic pathways. The most significantly enriched pathways included primary bile acid biosynthesis, cholesterol metabolism, and bile secretion. Other significantly enriched pathways included tryptophan metabolism, phosphatidylinositol signaling system, and various lipid metabolism pathways. The size of the circles in the plot indicates the count of differential metabolites involved in each pathway, with larger circles representing more metabolites. The significant enrichment of bile acid biosynthesis and cholesterol metabolism pathways in CX pigs is particularly interesting.

### 3.5. Intestinal Bacterial Diversity and Composition

**Figure 3:**
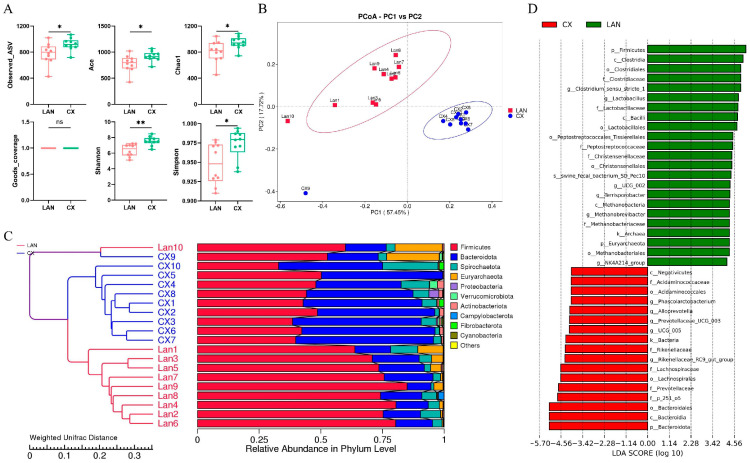
Intestinal bacterial diversity and composition in CX and LAN pigs. (**A**) Alpha diversity between CX and LAN pigs. (**B**) Principal Coordinate Analysis (PCoA) between CX and LAN pigs. (**C**) Unweighted Pair-group Method with Arithmetic Mean (UPGMA) between CX and LAN pigs. (**D**) LEfSe analysis between CX and LAN pigs. LAN, Landrace; CX, Congjiang Xiang. The asterisk indicates significant differences. * *p* < 0.05 ** *p* < 0.01.

To explore the colonic bacterial communities in CX and LAN pigs, 16S rRNA gene sequencing analysis was performed on colonic content samples, with results presented in Figure 3. The alpha diversity indices analysis revealed significant differences in bacterial diversity between the two breeds (Figure 3A). CX pigs exhibited significantly higher Observed_ASV, Ace, Chao, Shannon, and Simpson diversity indices compared to LAN pigs (*p* < 0.05), indicating greater microbial diversity and evenness in the colonic microbiota of CX pigs.

Principal Coordinate Analysis (PCoA) based on weighted UniFrac distances demonstrated clear separation between the microbial communities of CX and LAN pigs (Figure 3B). The samples from each breed formed distinct, non-overlapping clusters, suggesting fundamentally different microbial community structures between the two breeds. The first two principal coordinates explained a substantial portion of the total variance, with statistical analysis confirming significant differences in community structure between breeds (PERMANOVA, *p* < 0.01).

The Unweighted Pair-group Method with Arithmetic Mean (UPGMA) hierarchical clustering analysis (Figure 3C) further confirmed the distinct separation between CX and LAN pig bacteria. The dendrogram clearly showed that samples from the same breed clustered together, with a significant phylogenetic distance between the two breeds. The taxonomic composition analysis at the phylum level revealed that Firmicutes and Bacteroidota were the dominant phyla in both breeds, which is consistent with typical pig gut microbiota profiles. However, CX pigs showed significantly lower relative abundance of Firmicutes and higher abundance of Bacteroidota compared to LAN pigs (*p* < 0.05), resulting in a lower Firmicutes-to-Bacteroidota ratio (*p* < 0.05).

Linear discriminant analysis effect size (LEfSe) analysis (Figure 3D) identified the specific bacterial taxa that were significantly differentially abundant between CX and LAN pigs. CX pigs were characterized by significantly higher abundances of several beneficial bacteria, including *Rikenellaceae_RC9_gut_group*, *UCG_005*, *Prevotellaceae_UCG_003*, *Alloprevotella*, and *Phascolarctobacterium*. In contrast, LAN pigs showed higher *Clostridium_sensu_stricto_1*, *Lactobacillus*, *UCG _002*, *Terrisporobacter*, *Methanobrevibacter*, and *NK4A214_group*.

### 3.6. Intestinal Eukaryotic Microbial Diversity and Composition

**Figure 4:**
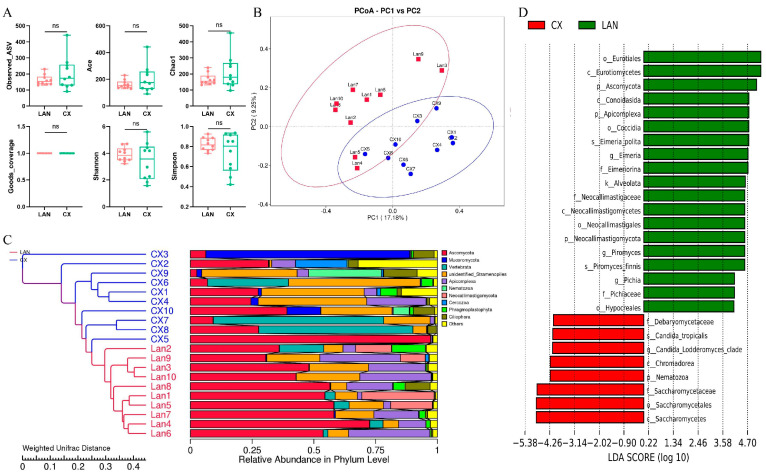
Intestinal eukaryotic microbial diversity and composition in CX and LAN pigs. (**A**) Alpha diversity between CX and LAN pigs. (**B**) Principal Coordinate Analysis (PCoA) between CX and LAN pigs. (**C**) Unweighted Pair-group Method with Arithmetic Mean (UPGMA) between CX and LAN pigs. (**D**) LEfSe analysis between CX and LAN pigs. LAN, Landrace; CX, Congjiang Xiang.

To explore the colonic eukaryotic microbial communities in CX and LAN pigs, 18S rRNA gene sequencing analysis was performed on colonic content samples, with results presented in Figure 4. The alpha diversity indices analysis revealed no significant differences in microbial diversity between the two breeds (Figure 4A). The PCoA based on weighted UniFrac distances demonstrated clear separation between the eukaryotic microbial communities of CX and LAN pigs (Figure 4B). The samples from each breed formed distinct, non-overlapping clusters, suggesting fundamentally different eukaryotic microbial community structures between the two breeds (PERMANOVA, *p* < 0.01).

The UPGMA hierarchical clustering analysis (Figure 4C) further confirmed the distinct separation between CX and LAN pig eukaryotic microorganisms. The dendrogram clearly showed that samples from the same breed clustered together, with a significant phylogenetic distance between the two breeds. The taxonomic composition analysis at the phylum level revealed that Ascomycota, mucoromycota, Vertebrata, unidentified_Stramenopiles, and Apicomplexa were the dominant phyla in both breeds. However, CX pigs showed significantly lower relative abundance of Ascomycota, Apicomplexa, and Neocallimastigomycota and higher Nematozoa compared to LAN pigs (*p* < 0.05).

The LEfSe analysis (Figure 4D) identified the specific eukaryotic microbial taxa that were significantly differentially abundant between CX and LAN pigs. CX pigs were characterized by significantly higher abundances of *Candida_Lodderomyces_clade*. In contrast, LAN pigs showed higher *Eimeria*, *Piromyces*, and *Pichia*.

### 3.7. Bacterial–Fungal Co-Occurrence Networks and Their Relationship with Muscle Flavor

**Figure 5:**
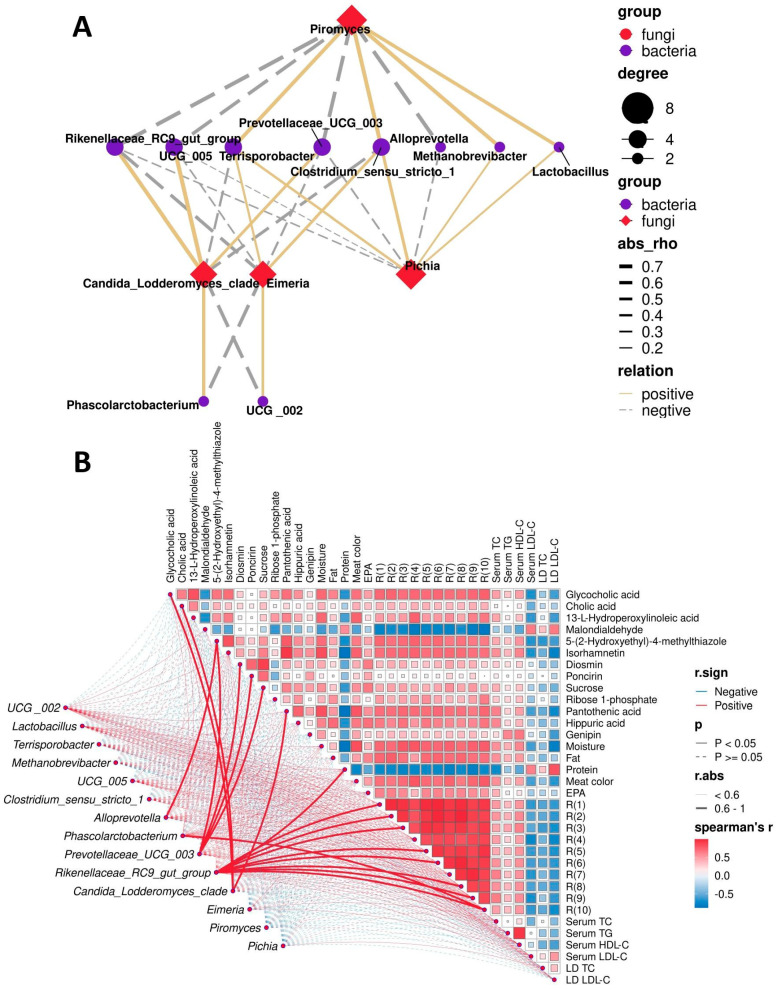
Bacterial–fungal co-occurrence networks and their relationship with muscle flavor compounds in CX and LAN pigs. (**A**) Bacterial–fungal interaction network showing correlations between microbial taxa. Purple circles represent bacterial taxa, red diamonds represent fungal taxa. Node size indicates the degree of connections. Solid orange lines represent positive correlations, while dashed gray lines represent negative correlations. (**B**) Correlation heatmap between microbial taxa and flavor compounds, meat quality parameters, and electronic nose sensor responses. Red indicates positive correlations, blue indicates negative correlations. The intensity of color represents the strength of correlation coefficients. LAN, Landrace; CX, Congjiang Xiang.

To investigate the relationships between gut microbiota, muscle metabolites, and meat quality parameters in CX and LAN pigs, correlation analyses were performed and are visualized in Figure 5. The correlation analysis revealed intricate relationship networks between intestinal bacterial–fungal interactions and muscle flavor formation.

The bacterial–fungal co-occurrence network analysis revealed important synergistic and antagonistic relationships (Figure 5A). *Rikenellaceae_RC9_gut_group* showed a strong positive correlation with the fungal taxon *Candida_Lodderomyces_clade* (r = 0.711, *p* < 0.001), while exhibiting a strong negative correlation with *Piromyces* (r = −0.714, *p* < 0.001). *UCG_005* similarly demonstrated a strong positive correlation with *Candida_Lodderomyces_clade* (r = 0.739, *p* < 0.001) and a negative correlation with *Piromyces* (r = −0.627, *p* < 0.01).

*Rikenellaceae_RC9_gut_group*, a bacterial taxon enriched in CX pigs, demonstrated strong positive correlations with multiple flavor-related compounds, including glycocholic acid (r = 0.238, *p* < 0.05), isorhamnetin (r = 0.575, *p* < 0.001), and pantothenic acid (r = 0.535, *p* < 0.001) (Figure 5B). More importantly, this bacterial group exhibited significant positive correlations with all electronic nose sensor responses (R1–R10, r values ranging from 0.434 to 0.691, *p* < 0.001), suggesting its crucial role in flavor development. Additionally, *Rikenellaceae_RC9_gut_group* showed positive correlations with meat moisture content (r = 0.531, *p* < 0.001) and a strong negative correlation with protein content (r = −0.609, *p* < 0.001).

*Phascolarctobacterium*, another bacterial taxon enriched in CX pigs, demonstrated similar correlation patterns with strong positive associations with glycocholic acid (r = 0.452, *p* < 0.001), cholic acid (r = 0.448, *p* < 0.05), isorhamnetin (r = 0.583, *p* < 0.001), and pantothenic acid (r = 0.538, *p* < 0.01). This bacterium also showed significant positive correlations with most electronic nose responses (R1–R10, r values ranging from 0.406 to 0.614, *p* < 0.05), particularly exhibiting the strongest correlation with the R10 sensor (r = 0.614, *p* < 0.01).

*Prevotellaceae_UCG_003*, as another characteristic bacterium of CX pigs, displayed strong positive correlations with key flavor compounds, particularly with 5-(2-hydroxyethyl)-4-methylthiazole (r = 0.863, *p* < 0.001), diosmin (r = 0.803, *p* < 0.001), poncirin (r = 0.866, *p* < 0.001), and sucrose (r = 0.858, *p* < 0.001), reaching the strong correlation range of 0.6–1.0.

*Candida_Lodderomyces_clade*, as a fungal taxon enriched in CX pigs, showed significant positive correlations with important flavor compounds, including glycocholic acid (r = 0.979, *p* < 0.05), cholic acid (r = 0.937, *p* < 0.05), isorhamnetin (r = 0.451, *p* < 0.01), and pantothenic acid (r = 0.610, *p* < 0.01). Conversely, certain microorganisms relatively enriched in LAN pigs showed negative correlations with flavor compounds. *Piromyces* exhibited negative correlations or non-significant correlations with most flavor compounds and electronic nose responses, consistent with its antagonistic role in the bacterial–fungal network.

*UCG_002* showed positive correlations with certain flavor compounds such as isorhamnetin (r = 0.392, *p* < 0.05) and pantothenic acid (r = 0.337, *p* < 0.01), but the correlation strengths were significantly lower than those of beneficial bacteria in CX pigs. Lactobacillus primarily showed positive correlation with protein content (r = 0.388, *p* < 0.001) and weak positive correlations with some electronic nose sensors, but non-significant correlations with major flavor compounds.

The correlation network analysis indicated that CX pigs formed a bacterial community centered on *Rikenellaceae_RC9_gut_group* and *Phascolarctobacterium* in their intestines, establishing synergistic relationships with fungi such as *Candida_Lodderomyces_clade* to collectively promote the production of flavor-related metabolites. Strong positive correlations (r > 0.4, *p* < 0.05) were observed between these microorganisms and key flavor compounds such as glycocholic acid, isorhamnetin, and pantothenic acid, as well as significant correlations with all electronic nose sensors.

## 4. Discussion

Breed-specific gut fungal and bacterial communities are strongly shaped by non-genetic factors—differential core microbiota among pig breeds are attributable to host genetics, diets, farms, and environments [18]—and that mycobiota composition is driven primarily by diet, nutrition, drug use, and physical condition [19]. We now explicitly state that observed CX/LAN differences likely reflect a combination of breed genetics, physiological maturity, diet, and rearing environment, and that our design cannot disentangle these, framing our findings within this context.

Indigenous pig breeds are valued for their superior meat quality, particularly their distinctive flavor characteristics. In the present study, CX pigs exhibited significantly higher moisture content and fat percentage compared to LAN pigs, which contribute to enhanced juiciness and flavor intensity. Chemically determined intramuscular fat (acid-hydrolyzed total lipid) includes membrane phospholipids and intracellular lipids that are not visible as marbling flecks, and subjective marbling scoring is a coarser, less sensitive measure than chemical quantification [20]. Thus, a modest chemical-fat difference need not manifest as a marbling-score difference—consistent with fat being a key but multi-form flavor substrate in meat [21].

Similarly, the meat color score was significantly higher in CX pigs, indicating a darker meat color that is generally preferred by consumers in many regional markets. These findings align with previous studies on indigenous breeds, where higher moisture content and intramuscular fat have been associated with improved sensory attributes [22]. The electronic nose analysis revealed significantly higher response values across all aroma parameters in CX pigs, particularly for sensors W1S/R(6), W2W/R(9), and W1W/R(7), which detect methane, organic sulfur compounds, and inorganic sulfur compounds, respectively. These compounds are critical contributors to meat flavor perception. Our findings are consistent with those reporting that indigenous pig breeds possess more complex and intense flavor profiles than commercial breeds [23,24]. The distinctive clustering of CX and LAN samples in the PCA plot further confirms the fundamental differences in flavor characteristics, which likely contribute to the premium market value of indigenous pork, as consumers increasingly seek products with enhanced sensory qualities [23].

The biochemical analysis revealed distinct lipid metabolism patterns between CX and LAN pigs that may contribute to their different meat quality and flavor characteristics. CX pigs exhibited significantly higher serum TC, HDL-C, and LDL-C, but lower TG levels compared to LAN pigs. These differences suggest more active cholesterol metabolism in CX pigs, potentially contributing to enhanced production of cholesterol-derived compounds that influence meat flavor. Interestingly, while serum LDL-C was higher in CX pigs, muscle LDL-C was significantly lower, indicating differential lipid partitioning between breeds. This finding aligns with Deng et al., who observed that indigenous breeds often demonstrate unique lipid metabolism patterns that favor intramuscular fat deposition without excessive subcutaneous fat accumulation [25]. The lower muscle LDL-C in CX pigs may contribute to improved oxidative stability during cooking, potentially reducing the formation of off-flavors caused by lipid oxidation. These metabolic differences likely reflect both genetic factors and gut microbiota influences, as recent studies have demonstrated that intestinal bacteria can modulate host lipid metabolism through multiple pathways, including bile acid transformation, short-chain fatty acid production, and regulation of lipid absorption [26,27]. The distinctive lipid metabolism profile of CX pigs provides further evidence of the complex physiological basis underlying their superior meat quality.

The untargeted metabolomics analysis identified 40 significantly differential metabolites in muscle tissue between CX and LAN pigs, with 27 up-regulated and 13 down-regulated in CX pigs, representing a comprehensive shift toward enhanced flavor-related metabolism. Key flavor compounds, including glycocholic acid, isorhamnetin, pantothenic acid, and 5-(2-hydroxyethyl)-4-methylthiazole, were substantially elevated in CX pigs. These compounds play crucial roles in meat flavor development: glycocholic acid and cholic acid are bile acids that regulate lipid digestion and metabolism, potentially influencing the formation of flavor precursors [28,29]; isorhamnetin, a flavonoid compound, contributes to antioxidant capacity and may protect flavor-sensitive compounds from oxidation [30]; and pantothenic acid (vitamin B5) is essential for CoA synthesis and fatty acid metabolism, directly linking to flavor compound formation [31]. The remarkable elevation of 5-(2-hydroxyethyl)-4-methylthiazole, a thiazole compound known for its roasted, nutty flavor characteristics, suggests enhanced Maillard reaction potential in CX pig meat [32]. KEGG pathway enrichment analysis revealed significant enrichment in bile acid biosynthesis, cholesterol metabolism, and bile secretion pathways, indicating enhanced lipid metabolism regulation that could influence intramuscular fat composition and flavor development [33,34]. Conversely, the down-regulation of malondialdehyde in CX pigs indicates superior antioxidant status, which helps preserve flavor compounds and meat quality during storage [35].

The gut bacterial diversity analysis revealed significantly higher alpha diversity indices in CX pigs compared to LAN pigs, indicating greater microbial diversity and evenness in the colonic microbiota of these indigenous pigs. This enhanced microbial diversity is likely to provide broader metabolic capabilities and resilience in the gut ecosystem, potentially contributing to improved nutrient utilization and metabolite production. Our findings are consistent with Ma et al. (2022), who reported higher gut microbiota diversity in indigenous pig breeds compared to commercial breeds [36]. The distinct clustering of samples in PCoA analysis confirmed fundamental differences in bacterial community structures between breeds. Notably, CX pigs exhibited a lower Firmicutes-to-Bacteroidota ratio compared to LAN pigs, which has been associated with improved metabolic health and reduced inflammation in previous studies [37]. The LEfSe analysis identified several bacterial taxa significantly enriched in CX pigs, including *Rikenellaceae_RC9_gut_group*, *Prevotellaceae_UCG_003*, *Alloprevotella*, and *Phascolarctobacterium*. These bacteria have been linked to fatty acid composition, dietary fiber degradation, short-chain fatty acid production, and amino acid metabolism in previous studies [38,39]. For instance, *Phascolarctobacterium* is known to produce propionate, which can influence host lipid metabolism and may indirectly affect meat flavor development [40]. The enrichment of these bacterial taxa in CX pigs suggests their potential role in contributing to the distinctive flavor profile of indigenous pork through their metabolic activities.

While fungal alpha diversity showed no significant differences between breeds, clear breed-specific clustering patterns were observed in the fungal communities of CX and LAN pigs. This finding highlights that breed is a major determinant of gut fungal composition in pigs, despite similar diversity indices. The distinct separation in both PCoA and UPGMA analyses suggests fundamentally different fungal community structures that may contribute to the different physiological and metabolic characteristics between breeds. CX pigs were characterized by significantly higher abundances of *Saccharomycetaceae*, *Debaryomycetaceae*, and *Candida_Lodderomyces_clade*, which are known to possess unique enzymatic capabilities. Saccharomycetaceae fungi, for instance, can produce various hydrolytic enzymes that aid in the breakdown of complex dietary components and may contribute to the production of flavor-related metabolites [41]. The enrichment of these fungal taxa in CX pigs represents a novel finding, as intestinal fungi have been largely overlooked in previous studies on meat quality determinants. As noted by Deng et al. (2025), fungi constitute an important yet often neglected component of the intestinal ecosystem with distinct metabolic capabilities that may influence host metabolism [42]. Our study is among the first to specifically examine fungal communities in relation to meat quality and flavor formation, providing new perspectives on the potential role of mycobiota in shaping meat characteristics in different pig breeds.

The correlation analysis between intestinal microbiota, muscle metabolites, and meat quality parameters revealed intricate relationships. *Rikenellaceae_RC9_gut_group*, which was more abundant in CX pigs, showed strong positive correlations with key flavor compounds, including glycocholic acid, isorhamnetin, and pantothenic acid, as well as with all electronic nose responses. Similarly, *Phascolarctobacterium* demonstrated positive associations with glycocholic acid and electronic nose responses. Notably, the fungal taxon *Piromyces* showed antagonistic relationships with these flavor-associated bacteria and negative correlations with flavor compounds and electronic nose responses. These findings suggest a complex interplay between bacteria and fungi in the gut that may influence meat flavor characteristics. The positive correlation between *Candida_Lodderomyces_clade* and flavor-associated bacteria further supports the concept of bacterial–fungal co-occurrence networks influencing meat quality. This multi-omics correlation approach represents a novel advancement in meat quality research, as previous studies have primarily focused on bacterial communities alone, neglecting potential bacterial–fungal interactions [43].

Based on our findings, we propose a mechanistic model of how bacterial–fungal interactions in the intestine influence meat flavor development in pigs. In CX pigs, the enrichment of beneficial bacteria such as *Rikenellaceae_RC9_gut_group* and *Phascolarctobacterium*, along with fungi like *Candida_Lodderomyces_clade*, creates a symbiotic network that enhances the production of flavor-related metabolites. These microorganisms likely engage in cross-feeding relationships, where the metabolic products of one species serve as substrates for others, leading to the generation of a diverse array of compounds that ultimately contribute to flavor. For instance, *Rikenellaceae* bacteria may produce short-chain fatty acids that influence host lipid metabolism, while specific fungi may metabolize complex polysaccharides to release precursors for flavor compound formation. Microbial metabolites such as amino acids, short-chain fatty acids, and bioactive compounds can be absorbed through the intestinal epithelium and transported to muscle tissue via the circulatory system, where they serve as precursors for flavor compounds formed during cooking or directly contribute to taste and aroma. Conversely, the antagonistic relationship between *Piromyces* and flavor-associated bacteria in LAN pigs may inhibit the production of beneficial metabolites, resulting in less distinctive flavor profiles. This proposed mechanism aligns with emerging evidence on the gut–muscle axis, which suggests that intestinal microbiota can significantly influence muscle metabolism through various signaling pathways [14,15]. The bacterial–fungal co-occurrence in CX pigs appears to create a favorable metabolic environment for the production of compounds that enhance meat flavor.

Several limitations should be acknowledged in the present study. First, while our correlation analysis demonstrates strong associations between gut microbiota and muscle flavor compounds, it does not establish direct causality. Controlled intervention studies involving microbiota transplantation or specific probiotic administration would be necessary to confirm causal relationships. Second, our sample size, although sufficient for detecting significant differences, may not capture the full range of variation within each breed. Third, potential confounding factors such as diet, environment, and age could influence both microbiota composition and meat quality parameters, although we attempted to minimize these by using standardized rearing conditions. Methodologically, the integration of multiple omics datasets presents challenges in data harmonization and interpretation, and our findings should be considered within the context of these analytical limitations. Additionally, fungal annotation and characterization remain challenging due to incomplete reference databases compared to bacterial databases, potentially limiting the depth of our fungal community analysis. Finally, while electronic nose analysis provides objective measures of aroma parameters, it may not fully capture the complexity of flavor perception as experienced by human consumers, and future studies would benefit from complementary sensory evaluation panels.

Future research should focus on establishing causal relationships between specific microbial taxa and meat flavor characteristics. Controlled intervention studies using gnotobiotic animal models or microbiota transplantation could provide direct evidence of causality. In vitro co-culture experiments with specific bacterial and fungal strains isolated from CX pigs would help elucidate the nature of their interactions and metabolic exchanges. Metabolic modeling approaches could be employed to predict the production of flavor compounds from microbial networks, providing a theoretical framework for targeted interventions. Additionally, longitudinal studies tracking changes in gut microbiota and meat quality parameters throughout the growth period could reveal critical developmental windows for microbial influence on flavor formation. The development of novel prebiotics or probiotics specifically targeting bacterial–fungal co-occurrence represents an exciting avenue for enhancing meat flavor in commercial breeds. Future studies should also explore the mechanisms by which microbial metabolites are transported from the gut to muscle tissue and their subsequent transformations during cooking. Integration of volatile compound analysis and consumer sensory evaluation would further strengthen the link between gut microbiota, muscle metabolites, and perceived flavor quality.

Our findings have several practical implications for pig production systems aiming to enhance meat quality. First, precision feeding strategies could be developed to selectively promote beneficial bacterial–fungal networks in the gut, potentially through the inclusion of specific dietary fibers or bioactive compounds that support the growth of *Rikenellaceae_RC9_gut_group*, *Phascolarctobacterium*, and *Candida_Lodderomyces_clade*. Second, microbiome-based breeding selection criteria could be established, where animals with favorable gut microbiota profiles are selected for breeding programs aimed at improving meat flavor. Third, traditional rearing practices of indigenous breeds, which have co-evolved with their distinctive microbiota over generations, could be analyzed to identify key environmental factors that promote beneficial microbial communities. These insights could then be incorporated into modified rearing systems for commercial breeds. Finally, novel probiotic formulations containing both bacterial and fungal components isolated from indigenous pigs could be developed specifically for enhancing meat flavor. Such microbial supplements could potentially confer some of the flavor benefits of indigenous breeds to commercial pigs, providing a practical approach to improving meat quality while maintaining production efficiency. These applications represent promising strategies for leveraging gut microbiota to enhance the sensory attributes of pork in a sustainable manner.

## 5. Conclusions

This study demonstrates that indigenous CX pigs differ from Landrace pigs in meat quality, odor/volatile profiles, muscle metabolites, and gut bacterial–fungal community structure. Correlation network analysis revealed strong positive associations between that specific taxa (*Rikenellaceae_RC9_gut_group*, *Phascolarctobacterium*, *Candida_Lodderomyces_clade*) and flavor-related metabolites such as glycocholic acid, isorhamnetin, and pantothenic acid, while antagonistic relationships were observed between *Piromyces* and flavor-associated bacteria. The enrichment of bile acid biosynthesis and cholesterol metabolism pathways in CX pigs further supported the role of microbial networks in flavor compound formation. Because these findings are correlational and the breeds differed in body weight, maturity, and potentially husbandry, the results identify candidate associations rather than causal mechanisms and should be confirmed in controlled interventional studies.

## Figures and Tables

**Table 1:** PEN 3.5 electronic nose sensor array and its performance characteristics.

Sensor No.	Sensor Name	Performance Characteristics
R(1)	W1C	Sensitive to aromatic compounds and benzene series
R(2)	W5S	Sensitive to nitrogen oxide compounds
R(3)	W3C	Sensitive to ammonia and aromatic compounds
R(4)	W6S	Sensitive to hydrogen
R(5)	W5C	Sensitive to alkane aromatic compounds
R(6)	W1S	Sensitive to short-chain alkanes such as methane
R(7)	W1W	Sensitive to inorganic sulfur compounds
R(8)	W2S	Sensitive to alcohols, ethers, aldehydes and ketones
R(9)	W2W	Sensitive to organic sulfur compounds
R(10)	W3S	Sensitive to long-chain alkane series

**Table 2:** Meat quality parameters in CX and LAN pigs ^a^.

Item	LAN	CX	*p*-Value
Moisture (%)	75.916 ± 0.784 ^b^	79.089 ± 1.293 ^a^	<0.001
Dry matter (%)	24.084 ± 0.502 ^a^	20.911 ± 0.836 ^b^	<0.001
Fat (% DM basis)	4.075 ± 0.033 ^b^	4.965 ± 0.838 ^a^	0.008
Protein (% DM basis)	93.470 ± 0.860 ^a^	90.680 ± 0.310 ^b^	0.042
pH_45min_	6.207 ± 0.148	6.339 ± 0.333	0.267
Meat color score	2.685 ± 0.520 ^b^	4.090 ± 0.475 ^a^	<0.001
Marbling score	1.750 ± 0.691	1.605 ± 0.286	0.548

^ab^ Within a row, means without a common superscript differ at *p* < 0.05. LAN, Landrace; CX, Congjiang Xiang.

**Table 3:** Flavor characteristics in CX and LAN pigs ^a^.

Item	LAN	CX	*p*-Value
W1C/R(1)	1.107 ± 0.008 ^b^	1.203 ± 0.051 ^a^	<0.001
W5S/R(2)	1.322 ± 0.037 ^b^	2.084 ± 0.446 ^a^	<0.001
W3C/R(3)	1.062 ± 0.002 ^b^	1.085 ± 0.013 ^a^	<0.001
W6S/R(4)	1.130 ± 0.013 ^b^	1.193 ± 0.026 ^a^	<0.001
W5C/R(5)	1.042 ± 0.001 ^b^	1.058 ± 0.009 ^a^	<0.001
W1S/R(6)	2.270 ± 0.188 ^b^	5.033 ± 1.618 ^a^	<0.001
W1W/R(7)	1.338 ± 0.084 ^b^	4.382 ± 1.985 ^a^	0.001
W2S/R(8)	1.552 ± 0.129 ^b^	3.391 ± 0.987 ^a^	<0.001
W2W/R(9)	1.604 ± 0.155 ^b^	4.804 ± 1.461 ^a^	<0.001
W3S/R(10)	1.126 ± 0.003 ^b^	1.152 ± 0.014 ^a^	<0.001

^ab^ Within a row, means without a common superscript differ at *p* < 0.05. LAN, Landrace; CX, Congjiang Xiang.

**Table 4:** Biochemical analysis of serum and LD muscle in CX and LAN pigs ^a^.

Item	LAN	CX	*p*-Value
Serum TC (mmol/L)	3.071 ± 0.334 ^b^	4.460 ± 1.068 ^a^	0.002
Serum TG (mmol/L)	1.441 ± 0.220 ^a^	1.075 ± 0.281 ^b^	0.004
Serum HDL-C (mmol/L)	4.488 ± 0.345 ^b^	5.608 ± 1.293 ^a^	0.024
Serum LDL-C (mmol/L)	1.951 ± 0.564 ^b^	2.786 ± 0.484 ^a^	0.002
LD TC (μmol/g)	7.249 ± 0.688	6.718 ± 0.628	0.089
LD TG (μmol/g)	6.496 ± 2.407	7.598 ± 2.527	0.332
LD HDL-C (μmol/g)	1.644 ± 1.331	1.746 ± 1.120	0.856
LD LDL-C (μmol/g)	0.898 ± 0.466 ^a^	0.276 ± 0.169 ^b^	0.002

^ab^ Within a row, means without a common superscript differ at *p* < 0.05. LAN, Landrace; CX, Congjiang Xiang.

**Table 5:** The differential flavor-related compounds of LD muscle in CX and LAN pigs ^a^.

No.	Compounds	VIP	*p*-Value	Log2(FC)	CX vs. LAN
1	Glycocholic acid	3.772	0.0002	8.962	up
2	Cholic acid	1.568	0.027	2.951	up
3	13-L-Hydroperoxylinoleic acid	2.137	0.007	1.808	up
4	Malondialdehyde	2.780	0.002	−1.788	down
5	Isorhamnetin	7.257	0.00000006	2.037	up
6	Diosmin	2.086	0.008	2.398	up
7	Poncirin	1.977	0.011	2.8702	up
8	5-(2-Hydroxyethyl)-4-methylthiazole	5.064	0.000009	6.043	up
9	Sucrose	1.784	0.016	2.405	up
10	Ribose 1-phosphate	1.385	0.041	1.053	up
11	Pantothenic acid	6.437	0.0000004	1.845	up
12	Hippuric acid	3.441	0.0004	2.133	up
13	Genipin	1.524	0.030	4.843	up

^a^ VIP = variable importance in the projection; VIP value of OPLS-DA (Orthogonal Partial Least Squares Discrimination Analysis); VIP > 1 means a significant difference. *p*-value of *t*-test; *p* < 0.05 means a significant difference. LAN, Landrace; CX, Congjiang Xiang.

## Data Availability

The original contributions presented in this study are included in the article. Further inquiries can be directed to the corresponding authors.
